# Frequency of HPV in oral cavity squamous cell carcinoma

**DOI:** 10.1186/s12885-018-4247-3

**Published:** 2018-03-27

**Authors:** Priscila Marinho de Abreu, Anna Clara Gregório Có, Pedro Leite Azevedo, Isabella Bittencourt do Valle, Karine Gadioli de Oliveira, Sônia Alves Gouvea, Melissa Freitas Cordeiro-Silva, Iúri Drummond Louro, José Roberto Vasconcelos de Podestá, Jeferson Lenzi, Agenor Sena, Elismauro Francisco Mendonça, Sandra Lúcia Ventorin von Zeidler

**Affiliations:** 1 Programa de Pós-Graduação em Biotecnologia, Centro de Ciências da Saúde, Universidade Federal do Espirito Santo, Vitoria, Espirito Santo Brazil; 2 Departamento de Patologia, Programa de Pós-graduação em Biotecnologia, Centro de Ciências da Saúde, Universidade Federal do Espirito Santo, Av. Marechal Campos, 1468 Maruípe, Vitória, ES 29.040-090 Brazil; 3 Departamento de Ciências Fisiológicas, Centro de Ciências da Saúde, Universidade Federal do Espirito Santo, Vitoria, Espirito Santo Brazil; 4 Faculdade Católica Salesiana do Espírito Santo, Vitória, Espírito Santo Brazil; 5 Programa de Prevenção e Detecção Precoce do Câncer Bucal, Setor de Cirurgia de Cabeça e Pescoço, Hospital Santa Rita de Cássia, Vitória, Espírito Santo Brazil; 6 Faculdade de Odontologia, Universidade Federal de Goiás, Goiânia, Goiás Brazil

**Keywords:** Oral cancer, HPV, Frequency, Incidence, Oral cavity, Squamous cell carcinoma, Human papillomavirus

## Abstract

**Background:**

The prevalence of high-risk human papillomavirus (HPV) DNA in cases of oral cavity squamous cell carcinoma (SCC) varies widely. The aim of this study is to investigate the frequency of high-risk HPV DNA in a large Brazilian cohort of patients with oral cavity SCC.

**Methods:**

Biopsy and resected frozen and formalin-fixed paraffin-embedded specimens of oral cavity SCC were available from 101 patients who were recruited at two Brazilian centres. Stringent measures with respect to case selection and prevention of sample contamination were adopted to ensure reliability of the data. Nested PCR using MY09/MY11 and GP5^+^/GP6^+^ as well as PGMY09/11 L1 consensus primers were performed to investigate the presence of HPV DNA in the tumours. HPV-positive cases were subjected to direct sequencing. Shapiro–Wilk and Student t test were used to evaluate data normality and to compare the means, respectively. Qualitative variables were analysed by logistic regression.

**Results:**

Our results demonstrate that the frequency of high-risk HPV types in oral cavity SCC is very low and is less than 4%. All HPV-positive cases were HPV16. In addition, our results do not show a significant association between the tumour clinical features and the risk factors (tobacco, alcohol and HPV) for oral cavity SCC.

**Conclusion:**

In the current study, we observed an overlapping pattern of risk factors that are related to tumour development. This, along with a low frequency of high-risk HPV DNA, supports the findings that HPV is not involved in the genesis of oral cavity SCC in Brazilian population.

## Background

Head and neck squamous cell carcinoma (HNSCC) is a significant cause of cancer morbidity worldwide as 650,000 new cases and 350,000 deaths occur every year [1]. HNSCC encompasses tumours of the oral cavity, oropharynx, hypopharynx and larynx, which are each associated with different risk factors and prognoses. Latin America has a relatively high incidence of these tumours [1]. It was estimated that 15,290 new cases of oral cavity squamous cell carcinoma (SCC) occurred in Brazil in 2015 [2]. The oral cavity is the most frequent site of cancer within the head and neck region and is in the “top ten” list of tumours with the highest incidences [1].

HNSCC is one of several cancers that is strongly associated with tobacco and alcohol use [3]. However, over the last 15 years, high-risk (HR) human papillomavirus (HPV) infection has also been aetiologically linked to a subset of HNSCCs. HPV involvement in oral and oropharyngeal carcinogenesis was first proposed by Syrjanen et al. in 1983 [4]. In 2007, the International Agency for Research on Cancer recognized human papillomavirus type 16 (HPV16) as the only carcinogenic type of HPV in sites other than the cervix uteri, including the anus, penis, vagina, vulva, oral cavity and oropharynx [5]. HPV16-associated carcinogenesis is mediated by expression of the viral E6 and E7 oncoproteins, which inactivate the tumour suppressor proteins p53 and retinoblastoma; this then disrupts cell cycle regulatory pathways [6]. Therefore, the lack of p53 mutations [7] and p16 protein accumulation [8–10], which occur as a result of the loss of transcriptional repression during early tumorigenesis, are considered to be hallmarks of HPV-related HNSCC [11].

Although the frequency of high-risk HPV that is detected may be higher in samples of HNSCC, substantial heterogeneity exists among studies in terms of the detection rates. A portion of this variation may be related to differences in the incidence among geographic locations and the head and neck subsites enrolled in the included studies [12]. Additionally, it is important to consider variations in the sample sources and the collection methods as well as in the HPV detection methods. Methods for the detection of HPV DNA include in situ hybridization and polymerase chain reaction (PCR), while methods for the detection of HPV E6/E7 RNA include quantitative reverse transcriptase PCR (qRT-PCR) and RNA in situ hybridization.

To support the involvement of HPV in head and neck tumours, few studies have been conducted to determine the frequency of HPV DNA exclusively in SCC of the oral cavity, specifically in Brazil [13, 14]. Most of the published studies have included multiple subsites of HNSCC, which have precluded the specific analysis of HPV involvement in oral carcinogenesis [15]. Furthermore, the frequency of HPV infection in oral cavity SCC exhibits much variation among studies worldwide as well in different regions within Brazil [13, 14, 16].

The aim of this study is to investigate the frequency of high-risk HPV in a large Brazilian cohort of patients with oral cavity SCC. The relevance of this study lies in our accuracy of the selection of tumour sites and in the collection of samples. We also used different HPV DNA detection methods and adopted stringent measures to prevent sample contamination, which ensures the reliability of the data.

## Methods

### Study subjects

This is a multicentre cross-sectional study conducted at the following two Brazilian centres: Santa Rita de Cassia Hospital and University Hospital Antonio Cassiano de Moraes, which are both located in Espírito Santo. This study was approved by the clinical centre ethics committees and by the National Commission on Ethics in Research (Protocols 318/2011 and 681/2011). Written consent was given by each patient prior to his or her participation in the study.

A total of 171 cases of oral cavity SCC that were diagnosed by histopathology between 2012 and 2015 were reviewed. The inclusion criteria included tumours from oral cavity that were diagnosed according to the International Classification of Diseases, version 10 [17] and patients who had not undergone any previous antineoplastic treatment. The anatomical subsites selected were: tongue (C02.0, C02.3, C02.8, C02.9); gum and alveolar ridge (C03.0, C03.1, C03.9); floor of the mouth (C04.0, C04.1, C04.8, C04.9); palate (C05.0, C05.8, C05.9); buccal mucosa (C06.0, C06.1); retromolar trigone (C06.2) and overlapping sites of other parts of mouth (C06.8, C06.9). Seventy cases were excluded for not presenting formalin-fixed paraffin-embedded or frozen tissues available.

Following patient consent, 82 fresh tumour specimens were collected at the time of biopsy or surgical resection into RNAlater® reagent (Qiagen, Valencia, CA, USA) and stored at − 80 °C until further processing. In addition, 19 formalin-fixed paraffin-embedded tumours were retrieved from the pathology archive at Santa Rita de Cassia Hospital.

Clinical and pathological data (i.e., age, gender, tumour site, TNM stage, alcohol consume and tobacco exposure) were obtained by interview and from the medical records. The clinical stage of the tumours was categorized as early (clinical stages I–II) or advanced clinical stages (clinical stages III–IV), and all tumours were classified according to the TNM classification system [18].

### DNA extraction and integrity

Genomic DNA was obtained from frozen tissues and from four 10 μm-thick sections cut from the paraffin blocks. Formalin-fixed paraffin-embedded sections were placed in 2 ml microcentrifuge tubes and dewaxed with multiple washes of xylene and graded solutions of 100%, 95% and 70% ethanol. After overnight digestion with sodium dodecyl sulphate (SDS) and 5% of 20 mg/ml of K proteinase (Sigma, Saint Louis, USA) at 37 °C, DNA was extracted from both types of samples by standard phenol-chloroform-isoamylic alcohol and sodium acetate-ethanol precipitation. Rigorous efforts were made to avoid cross-contamination at every stage. A new microtome blade was used each time a new case was sectioned, and the components of the microtome were cleaned with xylene and ethanol after each sample was sectioned. In addition, aerosol tips were used for all pipetting steps, and separate rooms were used for pre- and post-PCR experimental steps.

DNA concentrations were estimated using a DyNA Quant 200 Fluorometer (Hoeffer Scientific, Holliston, MA, USA). To exclude false negative results derived from the degradation of DNA in samples that were over-fixed, the integrity of the DNA was assessed by amplification of a fragment of the human β-globin gene using PCO3 (5’-ACACAACTGTGTTCACTAGC-3′) and PCO7 (5’-GAAAACATCAAGGGTCCCAT-3′) primers, which result in a 509-bp DNA fragment [19]. All β-globin-negative samples were excluded from further analysis.

### HPV DNA detection

#### Nested PCR using MY09/MY11 and GP5^+^/GP6^+^ primers

The detection of high-risk HPV was performed by nested PCR to amplify a part of the HPV L1 gene, which encodes the major capsid protein of several subtypes of HPV, as previously described by Jacob in 1995 [20]. Briefly, 50 ng of DNA from each sample was amplified with the consensus primers MY09/MY11 followed by amplification with general primers GP5^+^/GP6^+^ by two-step nested PCR. Standard PCR with the MY09/MY11 primers was performed as previously described [20, 21]. Each sample was amplified with 50 pmol each of the primers MY09 (5’-CGTCCMARRGGAWACTGATC-3′) and MY11 (5’-GCMCAGGGWCATAAYAATGG-3′) in the presence of 6 mM MgCl_2_ buffer, 200 mmol (each) dATP, dCTP, and dGTP, 600 mmol dUTP, and 7.5 U of Hot Start Taq DNA polymerase (Invitrogen, Waltham, MA, USA). Then, PCR was performed using the product of the first reaction as a template in 50 mM KCl, 10 mM TrisHCl (pH 8.3), 200 mM of each deoxynucleoside triphosphate, 3.5 mM MgCl_2_, 7.5 U of Hot Start Taq DNA polymerase (Invitrogen, Waltham, MA, USA), and 50 pmol each of the GP5^+^ (5’-TTTGTTACTGTGGTAGATACTAC-3′) and GP6^+^ (3’-CTTATACTAAATGTCAAATAAAAAG-5′) primers. Amplifications were performed in a Mastercycler Nexus (Eppendorf, Hamburg, DE) with an activation at 94 °C for 7 min and 25 cycles (first step) or 15 cycles (second step) at 94 °C for 45 s, 56 °C (first step) or 46 °C (second step) for 45 s and 72 °C for 1 min. This was followed by a final extension at 72 °C for 7 min, and storage at 4 °C.

#### PGMY09/11 L1 consensus PCR

Additionally, to ensure the reliability of the data, we subjected the samples to a second round of PCR using a consensus PGMY09/11 primer set. An equimolar mixture of each primer was added to the PCR master mix for a final concentration of 10 pmol of each oligonucleotide in the primer sets; the final MgCl_2_ concentration was 4 mM. Then, the PCR buffers, reagents, and amplification profiles were identical to those described by Gravitt in 2000 [21]. Cycling conditions were as follows: 95 °C for 5 min, followed by 30 cycles at 95 °C for 1 min, 57 °C for 1 min, 72 °C for 1 min and a 7-min final extension period at 72 °C (Mastercycler Nexus, Eppendorf, Hamburg, DE).

Positive and negative controls were used for each amplification and consisted of a previously known HPV-positive cervical carcinoma and ultrapure water, respectively. The products were then subjected to electrophoresis on an 8% polyacrylamide gel followed by a silver stain. Specimens were considered positive for HPV DNA using the MY09/MY11 and GP5^+^/GP6^+^ primer sets when they presented a 150-bp DNA fragment. This represents some of the low-risk genotypes (6, 11, 40, 42, 43, 44) or the high-risk genotypes (16, 18, 31, 33, 35, 39, 45, 51, 52, 56, 58, 59, 66, 68), as described by Jacobs (1995) [20]. Samples that were amplified using PGMY09/11 were considered HPV-positive if the PCR product was a 450-bp fragment. PGMY09/11 consensus primers allowed the detection of more than 30 HPV genotypes as follows: 6, 11,16, 18, 26, 31, 33, 35, 39, 40, 42, 45, 51, 52, 53, 54, 55, 56, 58, 59, 61, 62, 64, 66, 67, 68, 69, 70, 71, 72, 73, IS39, CP8304, CP6108, MM4, MM7, and MM8 [21].

#### DNA sequencing

PCR products were purified using the ExoSAP-IT PCR Clean-up Kit (GE Healthcare Life Sciences, Uppsala, SE) and were sequenced using the BigDye**®** Terminator v3.1 Cycle Sequencing Kit (Applied Biosystems, Foster City, CA, USA). Capillary electrophoresis was performed in an ABI Prism 310 Genetic Analyzer (Applied Biosystems, Foster City, CA, USA). The sequences were analysed using the Basic Local Alignment Search Tool (BLAST) available online at National Center for Biotechnology Information (https://blast.ncbi.nlm.nih.gov/Blast.cgi).

#### Statistical analysis

Shapiro–Wilk test was used to evaluate data normality. Variables distribution were presented by mean, standard deviation. Student t test was used to compare the mean of ages between HPV-positive and HPV-negative individuals. Multiple logistic regression was applied in order to compare HPV status, tumour size, and nodal involvement with risk factors. The level of significance adopted in all analysis was 5% with a 95% confidence interval. For the data analysis the Statistical Package for the Social Sciences, version 17 for Windows (SPSS, Chicago, USA) was used.

## Results

DNA from all 19 formalin-fixed paraffin-embedded specimens produced a β-globin PCR 509-bp fragment, as did the majority of the frozen specimens (71 of 82; 85.6%). DNA from 11 frozen biopsies which presented β-globin negative were excluded from the subsequent analysis. The reasons for the exclusion were the presence of degraded and low quality DNA from tiny fragments that could not be resubmitted to DNA extraction and the existence of PCR inhibitors. Thus, the HPV status was assessed in a total of 68 males and 22 females with a mean age at diagnosis of 57.9 years (range 30–93 years; standard deviation 12.2 years) and was reported as either positive or negative. The details concerning the clinical findings and the HPV status of the cases in our series are summarized in Table 1. Cases that exhibited HPV-positive status (3/90) were detected by nested PCR using the MY09/MY11 and GP5^+^/GP6^+^ primer sets, and the frequency of HPV infection was found to be 3.3%. Furthermore, 87 HPV-negative samples where enough DNA was present were also assessed by PGMY09/11; these samples presented a 100% concordance rate, which ensures the reliability of the previous results. The sequencing showed the presence of HPV16 in all HPV-positive cases originated from both formalin-fixed paraffin-embedded (1/19) and frozen tissues (2/71). No difference was observed considering the sample storage methods. Among the HPV-positive cases, two were heavy smokers and alcoholics males while the other was a female with no exposure to any of the known risk factors.

**Table 1:** Association of clinical findings and HPV status in oral cavity SCC (*n* = 90)

	*HPV*				*OR (Adjusted)*	*95% CI*	p-value**
	*Positive*		*Negative*				
	*n*	*(%)*	*n*	*(%)*			
Gender							
Male	2	(66.6)	66	(75.9)	1	–	–
Female	1	(33.4)	21	(24.1)	3.023	(0.135–67.544)	0.485
Tumour site							
Tongue	2	(66.7)	47	(54.0)	1,07E + 08	0	0.988
Floor of the mouth	0	(0.0)	22	(25.3)	1	–	–
Others^a^	1	(33.3)	18	(20.7)	2,07E + 08	0	0.998
Alcohol Consumption							
No	1	(33.3)	37	(43.0)	1	–	–
Yes	2	(66.7)	49	(57.0)	2.446	0	0.637
Tobacco Consumption							
No	1	(33.3)	39	(45.3)	1	–	–
Yes	2	(66.7)	47	(54.7)	1.993	(0.045–88.793)	0.722
TNM Stage							
I/II	0	(0.0)	28	(32.9)	1	–	–
III/IV	3	(100.0)	57	(67.1)	1,30E + 08	0	0.998

Reference category of the dependent variable - HPV negative

*OR* Odds Ratio, *CI* Confidence interval; **. Multiple logistic regression (Adjusted to all variables)

^a^Palate, retromolar trigone, gum, buccal mucosa, alveolar ridge

Data unknown: alcohol consumption - 1; tobacco consumption - 1; TNM stage - 2

All HPV-positive cases were in advanced stage of the disease (III-IV) presenting tumour size (T3/T4) and lymph node metastasis (N^+^) at the time of diagnosis. Besides that, there was no significant association among HPV status and TNM stage as well as gender, alcohol consumption, tobacco use or tumour site (Table 1). The mean age of HPV-positive patients was 61.0 years, whereas the mean age of the HPV-negative patients was 57.5 years; this difference was not significant (Table 2). In addition, the most used prognostic factors tumour size and nodal involvement were not associated with alcohol consumption, tobacco use, or HPV infection. Therefore, these variables could not be considered risk or protection factors in our cohort (Tables 2 and 3).

**Table 2:** Comparison of the ages of the patients with HPV-positive and HPV-negative oral cavity SCC

*HPV*	*n*	*Mean*	*STD* ^*b*^	*CI (95%)*	*p*
Negative	87	57.5	12.0	54.91–60.09	0.63^*a*^
Positive	3	61.0	16.0	46.00–79.00	

^a^*Student t* test

^b^*STD* Standard deviation

**Table 3:** Association of prognostic features and the risk factors (n = 90)

	*Tumour Size*				*OR (Adjusted)*	*95% CI*	p-value**	*Nodal Involvement* ^*a*^				*OR (Adjusted)*	*95% CI*	p-value**
	*T1/T2*		*T3/T4*					*N0*		*N+*				
	*n*	*(%)*	*n*	*(%)*				*n*	*(%)*	*n*	*(%)*			
HPV status														
Negative	36	(100.0)	48	(94.1)	1	–	–	48	(100.0)	36	(92.3)	1	–	–
Positive	0	(0.0)	3	(5.9)	1.18E + 09	0	0.999	0	(0.0)	3	(7.7)	2.04E + 09	0	0.999
Alcohol Drinkers														
No	16	(44.4)	21	(42.0)	1	–	–	21	(44.7)	16	(41.0)	1	–	–
Yes	20	(55.6)	29	(58.0)	0.708	(0.243–2.060)	0.526	26	(55.3)	23	(59.0)	0.626	(0.209–1.180)	0.404
Tobacco Users														
No	19	(52.8)	19	(38.0)	1	–	–	25	(53.2)	13	(33.3)	1	–	–
Yes	17	(47.2)	31	(62.0)	2173	(0.751–6.290)	0.152	22	(46.8)	26	(66.7)	2947	(0.977–8.887)	0.055

Reference category of the dependent variable Tumor size - T1/T2; Reference category of the dependent variable Nodal Involvement- N0; *OR* Odds Ratio

*CI* Confidence Interval; **. Multiple logistic regression (Adjusted to all variables);

^a^N0 - absence of lymph node metastasis; N+ − lymph node metastasis;

Data unknown: alcohol drinkers - 1; tobacco users - 1; tumour size - 3; nodal involvement - 3

## Discussion

This study presented a cohort with a significant number of patients with oral cavity SCC. Our results demonstrate that the frequency of high-risk HPV types in oral cavity SCC is very low and is less than 4%. We believe that this result is significant, as we have assessed a precise anatomical site in our cohort. We have thus avoided bias related to the differences in the incidence of oral SCC in other sites, especially in the oropharynx where the prevalence of high-risk HPV has been reported to be high [22, 23]. Tumour sites were validated by verification of the database and the medical records; in the three HPV-positive cases, the tumours were located in the tongue and in the alveolar ridge (Table 1). In addition, rigorous efforts were taken to prevent sample contamination as previously described, which ensures the consistency of our results.

The reported prevalence of high-risk HPV DNA in oral cavity SCC varies widely. A large multicentre study reported HPV DNA in 4% of 766 oral cancers using the consensus PCR primers GP5^+^/GP6^+^ [24]. Studies performed in United Kingdom and in the United States analysed large cohorts and found an HPV frequency of less than 2% and 5.6%, respectively [25, 26]. Moreover, in India, a study revealed an HPV infection rate of 46% in oral cavity SCC [27] while a meta-analysis published by Termine et al. (2008), in which 62 studies were analysed, revealed a 38.1% prevalence of HPV DNA in 4852 oral squamous cell carcinoma biopsy samples [28].

In Brazil, few studies have been conducted on the frequency of HPV in oral cavity SCC, and the data presented thus far are also discordant; data show a range of 0–19.2% frequency, which is mostly related to HPV16 infection [13, 16, 29]. Table 4 illustrates some of the consistent publications in recent years that have considered the frequency of HPV infection in oral cavity SCC.

**Table 4:** Prevalence of HPV in oral cavity SCC

Author / Year	Country	No. of cases	HPV (%)	Samples	Methods
Kaminagakura et al. [16] 2012	Brazil	114	19.2	FFPE	GP5^+^/GP6^+^
Attner et al. [40] 2011	Sweden	87	78.0	FFPE	GP5^+^/GP6^+^
Lopes et al. [25] 2011	United Kingdom	118	< 2.0	FT^b^/FFPE	GP5^+^/GP6^+^
Termine et al. [41] 2012	Italy	83	12.1	FFPE	Nested PCR^a^ PGMY09/11
Smith et al. [42] 2012	USA	170	9.4	FFPE	GP5^+^/GP6^+^ PGMY09/11
Lee et al. [34] 2012	Taiwan	173	38.0	FFPE	MY11/GP6^+^
Duray et al. [43] 2012	Belgium	147	44.2	FFPE	GP5^+^/GP6^+^
Lingen et al. [26] 2013	USA	409	5.9	FFPE	SPF10
González-Ramírez et al. [44] 2013	Mexico	80	5.0	FT^b^	Nested PCR^a^
Lopez et al. [13] 2014	Brazil	121	6.6	FT/FFPE	PGMY09/11
Chakrobarty et al. [27] 2014	India	83	46.0	FT^b^	MY09/MY11

^a^Nested PCR, MY09/11 and GP5^+^/GP6^+^

^b^*FT* frozen tissue

Thus, to evaluate the involvement of HPV in the genesis of oral cavity SCC, the geographic distribution of the populations should be considered. Besides, the wide variation in HPV detection rates can be influenced by other factors, such as sensitivity of the HPV testing method, the coverage of HPV genotypes in the test panel, sample collection methods as well as sample storage conditions.

The most widely applied HPV detection methods are based on the PCR amplification of viral DNA. In the current study, we have used three different standard primer sets (MY09/MY11, GP5^+^/GP6^+^ and PGMY09/11) and nested PCR to detect HPV DNA in head and neck tumours, which increases the sensitivity compared to a single PCR reaction [30]. Some advantages of the PCR methods used are the high sensitivity to detect more than 30 HPV genotypes, wide availability, and cost-effectiveness [31]. A weakness of the standard PCR-based assay is that it can not distinguish oncogenic virus from biologically irrelevant virus because is not possible to identify integrated or episomal DNA [25, 28, 32]. In addition, PCR techniques have lower specificity compared with in situ hybridization and are technically troublesome to perform.

Although it is expected that HPV detection rate by PCR is usually higher in frozen tissues, in our study we did not find difference when we compared it to formalin-fixed paraffin-embedded tumours. Thus, we suggest that the use of nested PCR, which was adopted to avoid interference of small fragments from DNA degradation during fixation procedures could contribute to this result. In addition, other studies have used both clinical specimens and have not observed differences in HPV DNA detection rates using PCR-based methods [13, 25]. Furthermore, Lopes et al., (2011) [25] report that non-quantitative PCR methods, when subject to stringent quality control measures, such as the methodology adopted in our study, are effective methods to detect HPV DNA in those samples.

Studies have reported that patients with oral cavity SCC who do not present with a history of alcohol and tobacco consumption tend to be younger than patients who smoke and drink alcohol [16, 33]. Thus, the low frequency of HPV DNA in the present study might be related to the high mean age of our cohort, as no difference was observed between the mean age of the individuals in the HPV-positive and HPV-negative groups. Moreover, most of our cases presented with an advanced stage of the disease and a history of long and intense exposure to known risk factors. Furthermore, no differences were observed among the anatomical subsites, tumour size, presence of lymph node metastasis and TNM stage among the HPV-positive and HPV-negative cases.

Furthermore, the frequency of HPV in our study is too close to the HPV DNA rates found in healthy individuals. The natural history of HPV infection has been extensively investigated in epidemiologic studies by PCR-based methods, HPV serology and DNA/RNA in situ hybridization [25, 34]. A review about epidemiological investigation on oral HPV prevalence in healthy individuals, published by Shigeishi & Sugiyama (2016), reported that HPV frequency in saliva of healthy individuals have shown low and variable rates in a period of time, which is related to each patient’s immune response and can, therefore, be inconstant. In addition, rates of oncogenic HPV infection in the oral cavity of healthy people are also known to be low (around 2%) and the natural history of HPV in this anatomical site shows HPV acquisition is a rare event compared to genital or anal infections [35, 36].

Data from a database in the USA shows a reduction in tobacco use over the last several decades [37]. Although HNSCC is closely linked to tobacco and alcohol use, its increasing incidence indicates that HPV16-related HNSCC arises in the oropharynx [24, 33, 38]. In contrast, the incidence of oral cavity SCC has declined significantly between 1973 and 2004 at a yearly rate of 1.85% [39], which suggests that HPV is not related to oral carcinogenesis. These results are reinforced by the variation in the frequency of HPV DNA, as demonstrated in this work and in other studies [40–44].

## Conclusion

In conclusion, in the current study, we observed an overlapping pattern of the risk factors tobacco and alcohol; this along with the low frequency of HPV DNA, supports the evidence that HPV is not involved in the development of oral cavity SCC in Brazilian population.

## Abbreviations

HNSCC: Head and Neck Squamous Cell Carcinoma
HPV: Human Papillomavirus
HPV16: Human Papillomavirus type 16
HR: High Risk
PCR: Polymerase Chain Reaction
qRT-PCR: Quantitative Reverse Transcriptase-PCR
SCC: Squamous Cell Carcinoma
SDS: Sodium dodecyl sulphate
